# Supplementary material for: Dynamic divisive normalization circuits explain and predict change detection in monkey area MT
Source: PLoS Comput Biol. 2021 Nov 12;17(11):e1009595. doi: 10.1371/journal.pcbi.1009595 (PMC8612546; doi:10.1371/journal.pcbi.1009595)
Supplement: S1 Data Code Repository — Running the code requires Matlab R2020a (The MathWorks, Inc.) and Python (Version 3.6). To perform all data analyses and fits, execute script ’PLOS_run_all.m’ from the main directory. See the comments in the scripts named ‘PLOS_run.m’ in the subdirectories for further information on the result plots and files created by running the code. (ZIP) [file pcbi.1009595.s001.zip › PLOS/Model/ICC/Color Profile EULA.pdf]

ADOBE SYSTEMS INCORPORATED

COLOR PROFILE LICENSE AGREEMENT

NOTICE TO USER: PLEASE READ THIS CONTRACT CAREFULLY. BY USING ALL OR ANY PORTION OF THE SOFTWARE YOU ACCEPT ALL THE TERMS AND CONDITIONS OF THIS AGREEMENT. IF YOU DO NOT AGREE WITH THE TERMS OF THIS AGREEMENT, DO NOT USE THE SOFTWARE.

1. DEFINITIONS In this Agreement, "Adobe" means Adobe Systems Incorporated, a Delaware corporation, located at 345 Park Avenue, San Jose, California 95110. "Software" means the software and related items with which this Agreement is provided.

2. LICENSE Subject to the terms of this Agreement, Adobe hereby grants you the worldwide, non-exclusive, nontransferable, royalty-free license to use, reproduce and publicly display the Software. Adobe also grants you the rights to distribute the Software only (a) as embedded within digital image files and (b) on a standalone basis. No other distribution of the Software is allowed; including, without limitation, distribution of the Software when incorporated into or bundled with any application software. All individual profiles must be referenced by their ICC Profile description string. You may not modify the Software. Adobe is under no obligation to provide any support under this Agreement, including upgrades or future versions of the Software or other items. No title to the intellectual property in the Software is transferred to you under the terms of this Agreement. You do not acquire any rights to the Software except as expressly set forth in this Agreement.

3. DISTRIBUTION If you choose to distribute the Software, you do so with the understanding that you agree to defend, indemnify and hold harmless Adobe against any losses, damages or costs arising from any claims, lawsuits or other legal actions arising out of such distribution, including without limitation, your failure to comply with this Section 3. If you distribute the Software on a standalone basis, you will do so under the terms of this Agreement or your own license agreement which (a) complies with the terms and conditions of this Agreement; (b) effectively disclaims all warranties and conditions, express or implied, on behalf of Adobe; (c) effectively excludes all liability for damages on behalf of Adobe; (d) states that any provisions that differ from this Agreement are offered by you alone and not Adobe and (e) states that the Software is available from you or Adobe and informs licensees how to obtain it in a reasonable manner on or through a medium customarily used for software exchange. Any distributed Software will include the Adobe copyright notices as included in the Software provided to you by Adobe.

4. DISCLAIMER OF WARRANTY Adobe licenses the Software to you on an "AS IS" basis. Adobe makes no representation as to the adequacy of the Software for any particular purpose or to produce any particular result. Adobe shall not be liable for loss or damage arising out of this Agreement or from the distribution or use of the Software or any other materials. ADOBE AND ITS SUPPLIERS DO NOT AND CANNOT WARRANT THE PERFORMANCE OR RESULTS YOU MAY OBTAIN BY USING THE SOFTWARE, EXCEPT FOR ANY WARRANTY, CONDITION, REPRESENTATION OR TERM TO THE EXTENT TO WHICH THE SAME CANNOT OR MAY NOT BE EXCLUDED OR LIMITED BY LAW APPLICABLE TO YOU IN YOUR JURISDICTION, ADOBE AND ITS SUPPLIERS MAKE NO WARRANTIES, CONDITIONS, REPRESENTATIONS OR TERMS, EXPRESS OR IMPLIED, WHETHER BY STATUTE, COMMON LAW, CUSTOM, USAGE OR OTHERWISE AS TO ANY OTHER

MATTERS, INCLUDING BUT NOT LIMITED TO NON-INFRINGEMENT OF THIRD PARTY RIGHTS, INTEGRATION, SATISFACTORY QUALITY OR FITNESS FOR ANY PARTICULAR PURPOSE. YOU MAY HAVE ADDITIONAL RIGHTS WHICH VARY FROM JURISDICTION TO JURISDICTION. The provisions of Sections 4, 5 and 6 shall survive the termination of this Agreement, howsoever caused, but this shall not imply or create any continued right to use the Software after termination of this Agreement.

5. LIMITATION OF LIABILITY IN NO EVENT WILL ADOBE OR ITS SUPPLIERS BE LIABLE TO YOU FOR ANY DAMAGES, CLAIMS OR COSTS WHATSOEVER OR ANY CONSEQUENTIAL, INDIRECT, INCIDENTAL DAMAGES, OR ANY LOST PROFITS OR LOST SAVINGS, EVEN IF AN ADOBE REPRESENTATIVE HAS BEEN ADVISED OF THE POSSIBILITY OF SUCH LOSS, DAMAGES, CLAIMS OR COSTS OR FOR ANY CLAIM BY ANY THIRD PARTY. THE FOREGOING LIMITATIONS AND EXCLUSIONS APPLY TO THE EXTENT PERMITTED BY APPLICABLE LAW IN YOUR JURISDICTION. ADOBE'S AGGREGATE LIABILITY AND THAT OF ITS SUPPLIERS UNDER OR IN CONNECTION WITH THIS AGREEMENT SHALL BE LIMITED TO THE AMOUNT PAID FOR THE SOFTWARE. Nothing contained in this Agreement limits Adobe's liability to you in the event of death or personal injury resulting from Adobe's negligence or for the tort of deceit (fraud). Adobe is acting on behalf of its suppliers for the purpose of disclaiming, excluding and/or limiting obligations, warranties and liability as provided in this Agreement, but in no other respects and for no other purpose.

6. TRADEMARKS Adobe and the Adobe logo are the registered trademarks or trademarks of Adobe in the United States and other countries. With the exception of referential use, you will not use such trademarks or any other Adobe trademark or logo without separate prior written permission granted by Adobe.

7. TERM This Agreement is effective until terminated. Adobe has the right to terminate this Agreement immediately if you fail to comply with any term hereof. Upon any such termination, you must return to Adobe all full and partial copies of the Software in your possession or control.

8. GOVERNMENT REGULATIONS If any part of the Software is identified as an export controlled item under the United States Export Administration Act or any other export law, restriction or regulation (the "Export Laws"), you represent and warrant that you are not a citizen, or otherwise located within, an embargoed nation (including without limitation Iran, Iraq, Syria, Sudan, Libya, Cuba, North Korea, and Serbia) and that you are not otherwise prohibited under the Export Laws from receiving the Software. All rights to use the Software are granted on condition that such rights are forfeited if you fail to comply with the terms of this Agreement.

9. GOVERNING LAW This Agreement will be governed by and construed in accordance with the substantive laws in force in the State of California as such laws are applied to agreements entered into and to be performed entirely within California between California residents. This Agreement will not be governed by the conflict of law rules of any jurisdiction or the United Nations Convention on Contracts for the International Sale of Goods, the application of which is expressly excluded. All disputes arising out of, under or related to this Agreement will be brought exclusively in the state Santa Clara County, California, USA.

10. GENERAL You may not assign your rights or obligations granted under this Agreement without the prior written consent of Adobe. None of the provisions of this Agreement shall be deemed to have been waived by any act or acquiescence on the part of Adobe, its agents, or employees, but only by an instrument in writing signed by an authorized signatory of Adobe.

When conflicting language exists between this Agreement and any other agreement included in the Software, the terms of such included agreement shall apply. If either you or Adobe employs attorneys to enforce any rights arising out of or relating to this Agreement, the prevailing party shall be entitled to recover reasonable attorneys fees. You acknowledge that you have read this Agreement, understand it, and that it is the complete and exclusive statement of your agreement with Adobe which supersedes any prior agreement, oral or written, between Adobe and you with respect to the licensing to you of the Software. No variation of the terms of this Agreement will be enforceable against Adobe unless Adobe gives its express consent, in writing, signed by an authorized signatory of Adobe.

ColorProfileDistributionLicense 03.13.03_4:39
