# Supplementary material for: Dynamic divisive normalization circuits explain and predict change detection in monkey area MT
Source: PLoS Comput Biol. 2021 Nov 12;17(11):e1009595. doi: 10.1371/journal.pcbi.1009595 (PMC8612546; doi:10.1371/journal.pcbi.1009595)
Supplement: S1 Data Code Repository — Running the code requires Matlab R2020a (The MathWorks, Inc.) and Python (Version 3.6). To perform all data analyses and fits, execute script ’PLOS_run_all.m’ from the main directory. See the comments in the scripts named ‘PLOS_run.m’ in the subdirectories for further information on the result plots and files created by running the code. (ZIP) [file pcbi.1009595.s001.zip › PLOS/Model/ICC/Profile Information.pdf]

Adobe

10/15/08

Adobe® ICC Profiles

Build quality color workflows with Adobe ICC profiles.

Adobe wants to enable consistent, high quality color workflows. For color workflows to succeed, color information must be shared by many people, from original creator to final publisher. For this reason we are supplying our ICC profiles as a free download for graphics professionals to use across their workflows.

Enclosed in our compressed archive are:

8 RGB profiles
Adobe RGB (1998)
Apple RGB
ColorMatch RGB
SMPTE-C
PAL/SECAM
HDTV (Rec. 709)
SDTV NTSC
SDTV PAL

14 CMYK profiles
US Web Coated (SWOP) v2
US Web Uncoated v2
Coated GRACoL 2006 (ISO 12647-2:2004)
Web Coated SWOP Grade 3 Paper
Web Coated SWOP Grade 5 Paper
Coated FOGRA27 (ISO 12647-2:2004)
Web Coated FOGRA28 (ISO 12647-2:2004)
Uncoated FOGRA29 (ISO 12647-2:2004)
Coated FOGRA39 (ISO 12647-2:2004)
Japan Web Coated (Ad)
Japan Color 2001 Coated
Japan Color 2001 Uncoated
Japan Color 2002 Newspaper
Japan Color 2003 Web Coated

NOTE: Users who have already installed recent versions of Adobe® Suites or certain Adobe® applications may not need to install these profiles. Many of these profiles are already included and installed when you install and update certain Adobe® applications.

For more information on ICC profiles, visit the ICC (International Color Consortium) web site, http://www.color.org/ .

For more information on ICC Color Management, see information on the Adobe web site, http://www.adobe.com/ .

Profile Installation

To install the Adobe ICC Profiles on Mac OS X:
You should install profiles in one of two locations.

Copy all of the ICC profiles to the /Users/<your login user name>/Library/ColorSync/Profiles folder. Profiles installed in this location will be available only to the user who installed the profiles.

or

Copy all of the ICC profiles to the /Library/ColorSync/Profiles folder. Profiles installed in this location require the user to be an administrator of the system. Profiles installed in this location will be available to all users.

To install the Adobe ICC Profiles on Windows 2000/XP & Vista:
Select the profiles in the "RGB Profiles" folder. While the profiles are selected, click and hold the right mouse button and choose the "Install Profile" menu item. Use this same procedure for the profiles in the "CMYK Profiles" folder. Profiles will be copied to the correct filesystem location and the Windows Registry will be updated. If the "Uninstall Profile" menu item appears you already have an Adobe ICC profile of the same name installed.

You may need to relaunch certain applications in order to access these profiles.

Reference Information for Adobe ICC Color Profiles

RGB Profiles

Adobe RGB (1998)

A color space with a larger gamut than sRGB IEC61966-2.1 but smaller than ProPhoto RGB. Good choice for print publication. Almost all colors available using standard offset press printing conditions can be encoded using this profile. Decodes color values to viewing room colorimetry. The specification for Adobe RGB (1998) can be found here: "http://www.adobe.com/digitalimag/adobergb.html". This is an output-referred, version 2 ICC profile.

Apple RGB

Describes the color characteristics of the legacy Apple Trinitron monitor, commonly used for press workflows before the widespread adoption of color management. This is an output-referred, version 2 ICC profile.

ColorMatch RGB

Describes the color characteristics of the Radius Pressview monitor, commonly used for press workflows before the widespread adoption of color management. This is an output-referred, version 2 ICC profile.

SMPTE-C

Color space based on color standards in the US television industry. Supersedes color definitions in the NTSC 1953 ICC profile. The specification for SMPTE-C can be found here: "http://www.smpte.org/smpte_store/standards/". This is an output-referred, version 2 ICC profile. SMPTE-C is the output-referred equivalent of the SDTV NTSC profile mentioned below.

PAL/SECAM

Color space based on color standards in the European television industry. The specification for PAL/SECAM can be found here: "http://www.ebu.ch/CMSimages/en/tec_doc_t3213_tcm6-10508.pdf". This is an output-referred, version 2 ICC profile. PAL/SECAM is the output-referred equivalent of the SDTV PAL profile mentioned below.

HDTV (Rec. 709)

Color space for high-definition video cameras. The specification for the ITU-R Recommendation 709 can be downloaded here: "http://www.itu.int/rec/R-REC-BT.709-5-200204-I/en". This is a camera-referred, version 4 ICC profile.

SDTV NTSC

Color space for NTSC video cameras. Uses the same tone response curve as that defined for HDTV (Rec. 709) specified in ITU-R Recommendation 709. This is a camera-referred, version 4 ICC profile.

SDTV PAL

Color space for PAL video cameras. Uses the same tone response curve as that defined for HDTV (Rec. 709) specified in ITU-R Recommendation 709. This is a camera-referred, version 4 ICC profile.

CMYK Profiles

NOTE: Gray balance and black start values are relative to paper white.

U.S. Web Coated (SWOP) v2

Characterization used: CGATS TR 001
URL for characterization: http://www.color.org/drsection1.html
K generation: light GCR
Black start: L*= 66, C = 40%
Max. K: 90%
Max. total ink: 300%
Paper White: L = 88.73, **a** = -0.27, **b** = 3.66
Gray Balance at 25% Cyan: C=25, M=20, Y=20
Gray Balance at 50% Cyan: C=50, M=41, Y=41
Gray Balance at 75% Cyan: C=75, M=68, Y=67

For additional information on the output conditions used to produce the CGATS TR 001 characterization, see http://www.npes.org/ or http://www.printtools.org/

U.S. Web Uncoated v2

Characterization used: GRACoL test sheet
URL for characterization: none
K generation: light GCR
Black start: L* = 67, C = 38%
Max. K: 95%
Max. total Ink: 260%
Paper White: L = 91.25, **a** = 0.48, **b** = -2.3
Gray Balance at 25% Cyan: C = 25, **M** = 18, **Y** = 17
Gray Balance at 50% Cyan: C = 50, **M** = 39, **Y** = 38
Gray Balance at 75% Cyan: C = 75, **M** = 62, **Y** = 63

Coated GRACoL 2006 (ISO 12647-2:2004)

Characterization used: CGATS TR 006
URL for characterization: http://www.color.org/drsection1.html
K generation: medium GCR
Black start: L* = 79, C = 26%
Max. K: 98%
Max. total Ink: 340%
Paper White: L = 95, **a** = -0.02, **b** = -1.96
Gray Balance at 25% Cyan: C = 25, **M** = 18, **Y** = 18
Gray Balance at 50% Cyan: C = 50, **M** = 40, **Y** = 40
Gray Balance at 75% Cyan: C = 75, **M** = 66, **Y** = 65

For additional information on GRACoL-recommended print output parameters, see http://www.gracol.org/

Web Coated SWOP Grade 3 Paper

Characterization used: CGATS TR 003
URL for characterization: http://www.color.org/drsection1.html
K generation: medium GCR
Black start: L* = 78, C = 26%
Max. K: 98%
Max. total Ink: 310%
Paper White: L = 92.5, **a** = 0, **b** = 0
Gray Balance at 25% Cyan: C = 25, **M** = 19, **Y** = 20
Gray Balance at 50% Cyan: C = 50, **M** = 41, **Y** = 41
Gray Balance at 75% Cyan: C = 75, M = 66, **Y** = 65

For additional information on SWOP-recommended print output parameters, see http://www.swop.org/

Web Coated SWOP Grade 5 Paper

Characterization used: CGATS TR 005
URL for characterization: http://www.color.org/drsection1.html
K generation: medium GCR
Black start: L* = 80, C = 24%
Max. K: 98%
Max. total Ink: 300%
Paper White: **L** = 90.06, **a** = -0.01, **b** = 4.14
Gray Balance at 25% Cyan: **C** = 25, **M** = 19, **Y** = 21
Gray Balance at 50% Cyan: **C** = 50, **M** = 41, **Y** = 43
Gray Balance at 75% Cyan: **C** = 75, **M** = 67, **Y** = 68

For additional information on SWOP-recommended print output parameters, see http://www.swop.org/

Coated FOGRA27 (ISO 12647-2:2004)

Characterization used: FOGRA27
URL for characterization: http://www.color.org/drsection1.html
K generation: light GCR
Black start: L* = 65, C = 45%
Max. K: 100%
Max. total Ink: 350%
Paper White: **L** = 95.97, **a** = 0.50, **b** = -3.30
Gray Balance at 25% Cyan: **C** = 25, **M** = 19, **Y** = 18
Gray Balance at 50% Cyan: **C** = 50, **M** = 40, **Y** = 37
Gray Balance at 75% Cyan: **C** = 75, **M** = 67, **Y** = 61

For additional information on the output conditions used to produce the FOGRA27 characterization, see http://www.fogra.org/

Web Coated FOGRA28 (ISO 12647-2:2004)

Characterization used: FOGRA28
URL for characterization: http://www.color.org/drsection1.html
K generation: light GCR
Black start: L* = 66, C = 41%
Max. K: 98%
Max. total Ink: 300%
Paper White: **L** = 92.37, **a** = -0.70, **b** = 1.52
Gray Balance at 25% Cyan: **C** = 25, **M** = 19, **Y** = 19
Gray Balance at 50% Cyan: **C** = 50, **M** = 40, **Y** = 38
Gray Balance at 75% Cyan: **C** = 75, **M** = 66, **Y** = 59

For additional information on the output conditions used to produce the FOGRA28 characterization, see http://www.fogra.org/

Uncoated FOGRA29 (ISO 12647-2:2004)

Characterization used: FOGRA29
URL for characterization: http://www.color.org/drsection1.html
K generation: light GCR
Black start: L* = 66, C = 40%
Max. K: 86%
Max. total Ink: 300%
Paper White: **L** = 95.71, **a** = 0.61, **b** = -2.32
Gray Balance at 25% Cyan: **C** = 25, **M** = 19, **Y** = 18
Gray Balance at 50% Cyan: **C** = 50, **M** = 41, **Y** = 39
Gray Balance at 75% Cyan: **C** = 75, **M** = 70, **Y** = 65

For additional information on the output conditions used to produce the FOGRA29 characterization, see http://www.fogra.org/

Coated FOGRA39 (ISO 12647-2:2004)

Characterization used: FOGRA39
URL for characterization: http://www.color.org/drsection1.html
K generation: medium GCR
Black start: L* = 86, C = 18%
Max. K: 330%
Max. total Ink: 98%
Paper White: **L** = 95, **a** = 0, **b** = -2
Gray Balance at 25% Cyan: **C** = 25, **M** = 18, **Y** = 19
Gray Balance at 50% Cyan: **C** = 50, **M** = 40, **Y** = 40
Gray Balance at 75% Cyan: **C** = 75, **M** = 65, **Y** = 64

For additional information on the output conditions used to produce the FOGRA39 characterization, see http://www.fogra.org/

Japan Color 2001 Coated

Characterization used: JC200103
URL for characterization: http://www.color.org/drsection1.html
Black start: L* = 49, C = 62%
Max. K: 80%
Max. total Ink: 350%
Paper White: **L** = 91.05, **a** = 0.28, **b** = -1.46
Gray Balance at 25% Cyan: **C** = 25, **M** = 19, **Y** = 18
Gray Balance at 50% Cyan: **C** = 50, **M** = 41, **Y** = 39
Gray Balance at 75% Cyan: **C** = 75, **M** = 69, **Y** = 65

For additional information on the output conditions used to produce the JC200103 characterization, see http://www.jpma-net.or.jp/

Japan Color 2001 Uncoated

Characterization used: JC200104
URL for characterization: http://www.color.org/drsection1.html
Black start: L* = 59, C = 42%
Max. K: 80%
Max. total Ink: 310%
Paper White: **L** = 92.32, **a** = 0.13, **b** = -0.32
Gray Balance at 25% Cyan: **C** = 25, **M** = 20, **Y** = 20
Gray Balance at 50% Cyan: **C** = 50, **M** = 40, **Y** = 45
Gray Balance at 75% Cyan: **C** = 75, **M** = 72, **Y** = 86

For additional information on the output conditions used to produce the JC200104 characterization, see http://www.jpma-net.or.jp/

Japan Web Coated (Ad)

Characterization used: JMPA provided print condition for DDCP (digital proof)
URL for characterization: http://www.j-magazine.or.jp/

For information on the output conditions used to produce the print characterization, see http://www.j-magazine.or.jp/

Japan Color 2002 Newspaper

Characterization used: JCN2002
URL for characterization: http://www.color.org/drsection1.html
Black start: L* = 65, C = 39%
Max. K: 95%
Max. total Ink: 240%
Paper White: **L** = 80.26, **a** = -0.24, **b** = 4.67
Gray Balance at 25% Cyan: **C** = 25, **M** = 20, **Y** = 22
Gray Balance at 50% Cyan: **C** = 50, **M** = 42, **Y** = 44
Gray Balance at 75% Cyan: **C** = 75, **M** = 73, **Y** = 72

For additional information on the output conditions used to produce the JCN2002 characterization, see http://www.jpma-net.or.jp/

Japan Color 2003 Web Coated

Characterization used: JCW2003
URL for characterization: http://www.color.org/drsection1.html
Black start: L* = 52, C = 57%
Max. K: 80%
Max. total Ink: 320%
Paper White: L = 92.3, **a** = -0.51, **b** = 0.03
Gray Balance at 25% Cyan: C = 25, **M** = 18, **Y** = 19
Gray Balance at 50% Cyan: C = 50, **M** = 38, **Y** = 38
Gray Balance at 75% Cyan: C = 75, **M** = 67, **Y** = 65

For additional information on the output conditions used to produce the JCW2003 characterization, see http://www.jpma-net.or.jp/
