# Supplementary material for: Dynamic divisive normalization circuits explain and predict change detection in monkey area MT
Source: PLoS Comput Biol. 2021 Nov 12;17(11):e1009595. doi: 10.1371/journal.pcbi.1009595 (PMC8612546; doi:10.1371/journal.pcbi.1009595)
Supplement: S1 Data Code Repository — Running the code requires Matlab R2020a (The MathWorks, Inc.) and Python (Version 3.6). To perform all data analyses and fits, execute script ’PLOS_run_all.m’ from the main directory. See the comments in the scripts named ‘PLOS_run.m’ in the subdirectories for further information on the result plots and files created by running the code. (ZIP) [file pcbi.1009595.s001.zip › PLOS/Model/ICC/Trademark Information.pdf]

Adobe®

5/10/05

Trademark Information

Adobe is either a registered trademark or trademark of Adobe Systems Incorporated in the United States and/or other countries. All instances of the name "Adobe RGB" are references to the Adobe RGB (1998) color space and color encodings as defined by Adobe, unless otherwise stated. The name "Adobe RGB (1998)" also is used as a software product trademark for Adobe's implementation of the Adobe RGB (1998) ICC profile. Adobe does not permit the use of the Adobe RGB trademark for software, hardware, or other related products from companies other than Adobe, unless the company has obtained a prior written license from Adobe to do so.

Distributing the Adobe RGB (1998) ICC profile

You may distribute the Adobe RGB (1998) ICC profile as embedded within digital image files and on a standalone basis so long as you accept the terms and conditions of the Color Profile License Agreement on this page and the Adobe end-user license agreement.

Keep in mind that you must sign a supplementary license agreement with Adobe if you are interested in distributing the Adobe RGB (1998) ICC profile software embedded or as a bundle with a camera, display or other hardware device or software application. If you have not already signed an agreement, please contact your Adobe representative.
